# The dilemma of food genetics and improvement

**DOI:** 10.1515/biol-2025-1150

**Published:** 2025-08-02

**Authors:** Mohamed Zarid

**Affiliations:** Laboratory of Botany, Biotechnology and Plant Protection, Faculty of Science, Ibn Tofail University, BP 242, Kenitra, Morocco

**Keywords:** crop improvement, nutrients, biofortification, food authenticity, health risks

## Abstract

The advancement of genetic techniques in food production offers significant potential for improving crop quality, yield, and sustainability. However, these innovations present a complex set of dilemmas concerning nutritional quality, food authenticity, socioeconomic implications, regulatory challenges, and human health. This article explores contemporary genetic methods such as marker-assisted selection, genome editing (CRISPR-Cas9), and tissue culture, highlighting their applications and limitations. Additionally, transgenic approaches and biofortification strategies are examined for their role in enhancing nutritional value. It examines the trade-offs between organoleptic properties and nutritional integrity, revealing a concerning trend toward prioritizing sensory attributes over health benefits. Furthermore, the potential health implications of genetic manipulation, including links to autoimmune and hormonal disorders, are critically analyzed with a focus on allergenicity concerns and long-term safety assessments. The discussion underscores the urgent need for sustainable practices in food genetics that align with environmental goals and public health priorities. Finally, a call to action is made for collaborative dialogue among scientists, policymakers, and consumers to prioritize health, sustainability, and transparency in food production, ensuring that agricultural advancements benefit all stakeholders while preserving the ecological balance for future generations.

## Graphical abstract

**Figure:**
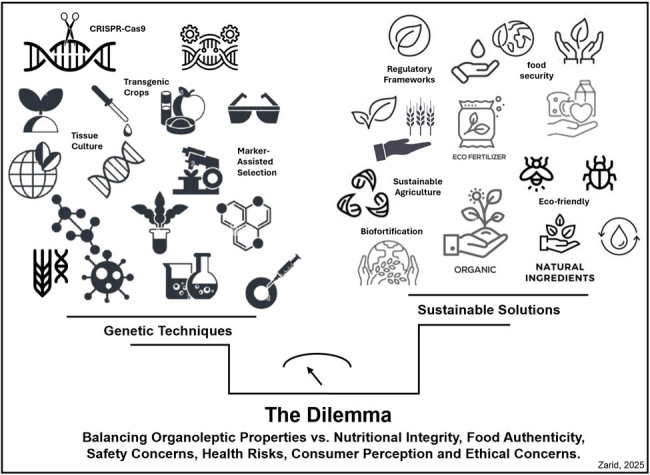

## 1 Introduction

Contextual background: Food genetics and improvement have become pivotal in addressing the challenges faced by contemporary agriculture. As the global population continues to grow – expected to reach 9.7 billion by 2050 – there is an urgent need to enhance food production systems to ensure food security and sustainability [1]. Traditional agricultural practices are increasingly strained by climate change, soil degradation, and the rising demand for nutritious food. In response, genetic techniques, including marker-assisted selection (MAS), genome editing (such as CRISPR-Cas9), transgenic modifications, and biofortification, have emerged as critical tools for improving crop resilience, yield, and quality [2,3].

These techniques enable the introduction of desirable traits such as disease resistance, drought tolerance, and improved nutritional profiles, thus playing an important role in developing more sustainable food systems [4]. Moreover, the potential for precision agriculture has opened new avenues for reducing the environmental impact of farming, as these technologies can help optimize inputs and minimize waste [5]. However, the rapid advancement of genetic technologies also brings forth complex ethical, social, economic, regulatory, and health-related dilemmas that must be carefully navigated. Concerns surrounding consumer perception, allergenicity, and long-term safety assessments further complicate their widespread acceptance.

Thesis statement: The central argument of this article is that while genetic techniques in food production hold promise for enhancing agricultural sustainability and addressing food security, they also pose significant dilemmas regarding the authenticity of food products, their nutritional value, socioeconomic consequences, regulation frameworks, and potential health implications. The reliance on these technologies raises critical questions about the balance between improving crop yield and quality versus preserving the nutritional integrity and authenticity of our food supply. As we explore the implications of these advancements, it becomes essential to consider how they impact not only the food we consume but also the long-term health of individuals and ecosystems alike [6,7]. Furthermore, assessing their effects on global trade, farmer livelihoods, and consumer trust is imperative for equitable and sustainable food systems.

## 2 Current genetic techniques in crop improvement

### 2.1 MAS

MAS is a powerful breeding tool that utilizes molecular markers to identify and select plants with desirable traits. This technique involves the identification of specific DNA sequences associated with traits of interest, allowing breeders to select individuals with the desired genetic characteristics without relying solely on traditional phenotypic evaluation. The process typically starts with mapping the genome of the target species to identify markers linked to important traits such as disease resistance, drought tolerance, or yield potential.

The benefits of MAS are manifold. It accelerates the breeding process by enabling the selection of superior genotypes at the seedling stage, thereby reducing the time and resources needed to develop new cultivars [2]. Moreover, MAS can enhance the genetic diversity of crops, which is essential for resilience against environmental stresses. For example, Abdul Aziz et al. [2] highlight the application of MAS in developing disease-resistant varieties of staple crops, which contributes to sustainable agricultural systems by reducing reliance on chemical pesticides and improving food security (Table 1).

**Table 1:** Comparison of conventional breeding, MAS, and molecular breeding

Criteria	Conventional breeding	MAS	Molecular breeding (e.g., CRISPR, RNAi)
Precision	Low – relies on phenotype and linkage drag	Moderate – uses genetic markers to improve accuracy	High – targets specific genes or nucleotides
Speed	Slow – requires multiple generations	Faster – early selection possible using markers	Fast – direct edits reduce breeding cycles
Trait specificity	Broad, polygenic traits	Trait-specific if markers are well-characterized	Highly specific, gene-level modifications possible
Genetic predictability	Low – segregation may mask traits	Improved – linked markers offer predictability	High – direct and heritable edits
Cost	Low upfront but labor-intensive	Moderate – requires molecular infrastructure	High initially, but cost-effective in the long run
Regulatory complexity	Low – no foreign DNA introduced	Low to moderate – dependent on species and markers	High – especially for transgenic edits; evolving for CRISPR
Public acceptance	High – traditional method	Moderate – viewed as conventional enhancement	Variable – dependent on technology and region
Use cases	Landrace improvement, hybrid vigor	Disease resistance, yield improvement	Drought tolerance, biofortification, shelf-life extension

### 2.2 Genome editing (CRISPR-Cas9)

Genome editing, particularly through the CRISPR-Cas9 technology, represents a significant advancement in the precision of genetic modifications. This technique allows for targeted changes to an organism’s DNA, enabling the addition, deletion, or alteration of specific gene sequences. The CRISPR system consists of two key components: the Cas9 enzyme, which acts as molecular scissors to cut the DNA, and a guide RNA that directs the Cas9 to the specific location in the genome.

The potential of CRISPR-Cas9 in crop improvement is vast. It facilitates the development of varieties with enhanced traits such as improved nutritional content, increased resistance to biotic and abiotic stresses, and enhanced yield [3]. For instance, Jaganathan et al. [8] provide examples of successful CRISPR applications in crops like rice and wheat, where targeted gene edits have led to significant improvements in agronomic traits. The precision of this technology minimizes unintended effects compared to traditional genetic modification, making it a valuable tool in modern agriculture. Examples include bioengineered wheat with higher protein content and rice varieties with increased vitamin A levels, contributing to global nutrition. Recent advancements in CRISPR-based precision breeding have led to significant improvements in crop traits. Li et al. [9] demonstrated enhanced grain yields in rice and wheat through precise genome editing, targeting genes responsible for cytokinin degradation. Additionally, CRISPR-mediated disease resistance has been successfully applied to cucumbers and tomatoes, offering improved resilience against viral and fungal infections [10]. Furthermore, Xu et al. [11] utilized CRISPR technology to increase sugar content in tomatoes without compromising fruit size, highlighting its potential for tailoring crop organoleptic properties.

#### 2.2.1 Emerging approaches: base editing, prime editing, and synthetic biology

Recent innovations have extended the capabilities of genome editing beyond CRISPR-Cas9 through base editing and prime editing, which allow precise, programmable changes to DNA without introducing double-strand breaks. Cytosine and adenine base editors have been used to induce point mutations conferring herbicide resistance in rice by targeting the *ALS* gene, demonstrating high efficiency and specificity [12]. Prime editing, which uses a reverse transcriptase fused to a Cas9 nickase, enables the insertion, deletion, or replacement of short DNA sequences. Li et al. [13] successfully used this technique in tomato to modify ripening-related genes, improving shelf life and flavor traits without foreign DNA integration.

In parallel, synthetic biology platforms are emerging as transformative tools in crop biotechnology. These platforms involve the modular design of genetic circuits to control gene expression, metabolic pathways, or stress responses. Engineered microbial chassis have been used to produce plant metabolites such as resveratrol, while in planta synthetic systems are being developed for nutrient biofortification and inducible gene expression [14,15]. These approaches provide a flexible and programmable toolkit for future trait engineering, potentially simplifying regulatory evaluation due to the absence of transgenes or reliance on endogenous pathways.

### 2.3 Tissue culture and somaclonal variation

Tissue culture is a technique that involves growing plant cells or tissues in a controlled, sterile environment. This method is widely used for the rapid propagation of plants, allowing for the production of disease-free and genetically uniform plantlets. In addition to its role in micropropagation, tissue culture can also facilitate the study of somaclonal variation, which refers to genetic variations that occur in plants regenerated from tissue culture.

Somaclonal variation can lead to the emergence of new traits that may not be present in the original plant, providing an additional source of genetic diversity for breeders [13]. This technique is particularly beneficial for the improvement of horticultural crops, as it allows for the selection of variants with desirable traits such as increased yield, better fruit quality, or enhanced disease resistance. Krishna et al. [20] discuss various applications of somaclonal variation in horticultural crops, demonstrating its potential for generating improved cultivars through innovative breeding strategies. Moreover, tissue culture plays a critical role in developing virus-free planting material, especially for crops such as bananas and potatoes, ensuring improved crop productivity.

### 2.4 Transgenic approaches in crop improvement

Transgenic modification typically involves the introduction of genes from another species to confer desirable traits. In contrast, CRISPR and related genome-editing technologies predominantly induce precise modifications within the plant’s own genome, without adding foreign genes.

One of the most successful applications of transgenic technology is the introduction of *Bacillus thuringiensis* (Bt) genes into crops like cotton and maize. Bt crops have significantly reduced pesticide use, contributing to environmental sustainability while improving yields. Additionally, transgenic techniques have been used to develop herbicide-resistant crops, allowing for better weed management and reduced soil degradation.

Despite these benefits, public skepticism and regulatory hurdles remain significant challenges for transgenic crops. Concerns over potential allergenicity, gene flow to wild relatives, and long-term ecological effects continue to fuel debates on their widespread adoption. Regulation frameworks vary across countries, with the European Union maintaining stringent approval processes compared to more permissive policies in the United States and Brazil [4]. Future research must address these concerns through improved safety assessments and transparent communication with consumers.

### 2.5 Advanced molecular mechanisms in crop improvement

Beyond conventional genetic tools, several advanced molecular mechanisms now enable targeted and efficient trait development in crops.

#### 2.5.1 CRISPR/Cas9 technology

CRISPR-Cas9, derived from bacterial immune systems, allows site-specific genome editing via the Cas9 nuclease guided by a short RNA molecule. This technology enables knockout or modification of target genes, facilitating precise trait development. For example, in low-gluten wheat, CRISPR has been used to disrupt *gliadin* gene clusters, reducing immunogenic proteins responsible for triggering celiac disease [8]. Additionally, Xu et al. [11] demonstrated the successful knockout of genes involved in sugar metabolism in tomatoes to enhance sweetness without compromising yield.

#### 2.5.2 RNA interference (RNAi)

RNAi is a post-transcriptional gene-silencing mechanism that utilizes double-stranded RNA to degrade homologous messenger RNAs, thus preventing protein synthesis. It has been applied to reduce allergens or pest susceptibility in crops. RNAi-based maize varieties, for instance, silence essential genes in corn rootworm larvae upon ingestion, effectively reducing pest populations without chemical pesticides [3].

#### 2.5.3 Promoter engineering

This technique involves modifying regulatory sequences to fine-tune gene expression. Tissue-specific or inducible promoters can drive precise expression of transgenes. In Golden Rice, the *psy* and *crtI* genes were expressed under endosperm-specific promoters to ensure β-carotene production only in edible grain parts, minimizing off-target expression and metabolic burden [9].

#### 2.5.4 Gene stacking

Gene stacking combines multiple desirable traits into a single variety, often through successive transformation or breeding strategies. In Bt cotton, for example, the *Cry1Ac* and *Cry2Ab* genes are stacked to confer broader-spectrum resistance against multiple Lepidopteran pests, reducing pesticide dependence and increasing yield [4]. This method has also been applied in maize and soybean to combine herbicide tolerance with insect resistance, showcasing its versatility.

**Figure 1:**
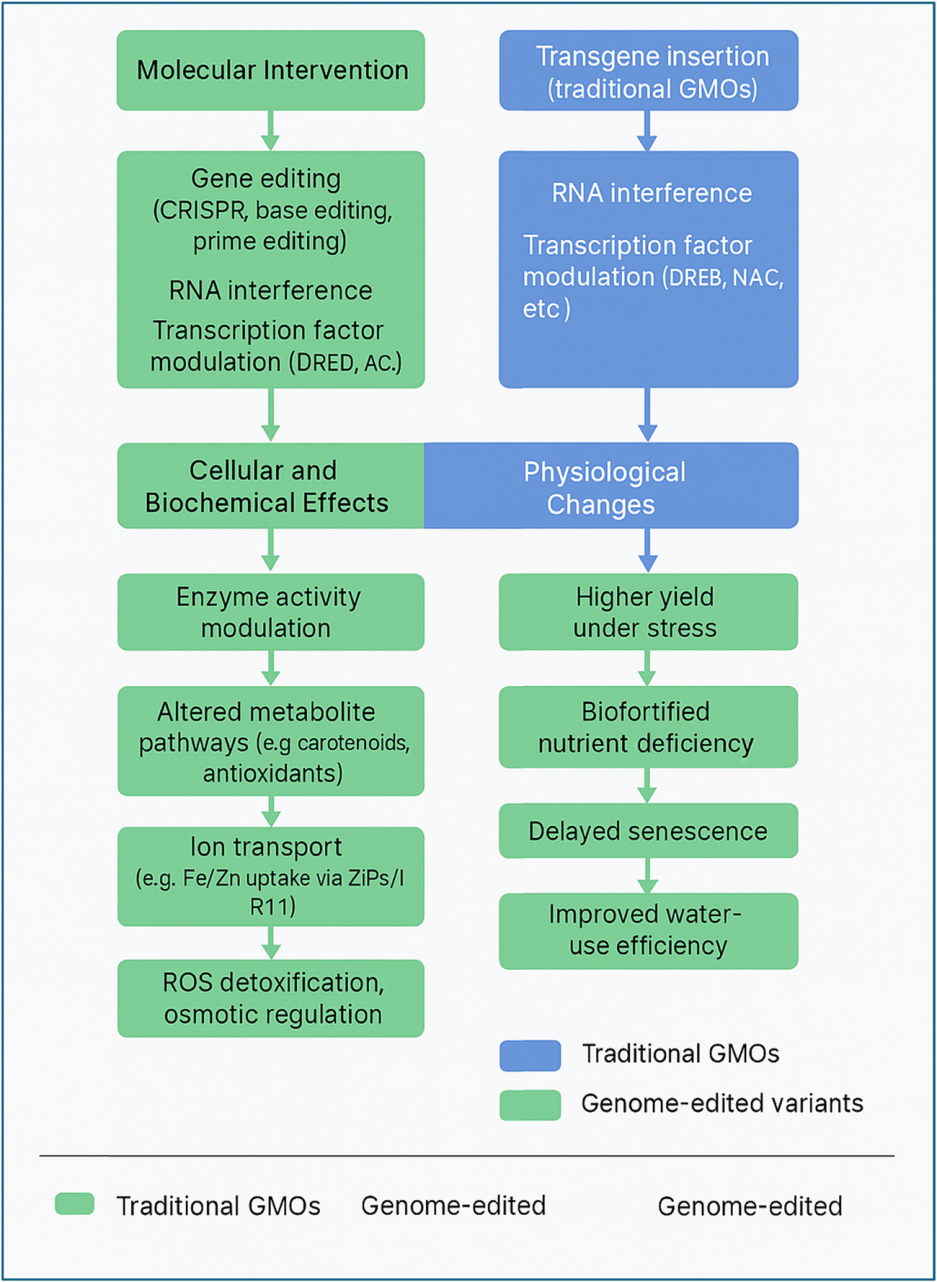
Molecular interventions and their impact on crop traits and agronomic performance.

These molecular approaches enhance the precision, efficiency, and predictability of trait development, marking a shift from random mutagenesis or classical selection toward rational, mechanism-based crop design (Figure 1).

## 3 Nutritional quality vs organoleptic properties

### 3.1 Trade-offs in selection

In contemporary agriculture, the selection of crop varieties often prioritizes organoleptic properties – those characteristics that affect the senses, such as taste, texture, color, and aroma – over nutritional quality. This emphasis is driven by market demands, as consumers frequently favor visually appealing and flavorful produce that enhances their culinary experiences. However, this focus can come at a significant cost to the nutritional value of the food produced.

Tapsell et al. [21] highlight the essential role of nutritional components in maintaining health and preventing chronic diseases. While the organoleptic qualities of fruits and vegetables can enhance consumer enjoyment and marketability, the potential trade-offs include a decline in essential nutrients such as vitamins, minerals, and phytochemicals. For example, breeding practices that enhance sweetness and visual appeal may inadvertently reduce levels of beneficial compounds like antioxidants and dietary fiber. This phenomenon raises important questions about the long-term implications for public health, as nutrient deficiencies can contribute to a variety of health issues, including obesity and metabolic disorders.

Moreover, the challenge lies in balancing the dual goals of improving organoleptic properties while simultaneously enhancing the nutritional profile of crops. Innovations in breeding strategies, such as the integration of traditional breeding with modern genetic techniques, can help to address these trade-offs by focusing on traits that enhance both flavor and nutrition [21]. Advanced genome editing techniques, such as CRISPR-Cas9, offer potential solutions by targeting specific genes responsible for both sensory and nutritional attributes, minimizing trade-offs while optimizing health benefits.

### 3.2 Nutritional enhancement via biofortification

Biofortification aims to increase micronutrient content in staple crops through genetic modification. This strategy is particularly valuable for addressing global malnutrition, as it enhances the nutritional profile of widely consumed crops without requiring significant dietary changes.

At the molecular level, biofortification strategies often involve the overexpression of metal transporters such as *OsIRT1* and *OsZIP1* for enhanced iron and zinc uptake, and ferritin for improved storage capacity. Transcriptional regulators like *OsNAS2* (nicotianamine synthase) have been shown to increase metal chelation and phloem mobility, contributing to improved micronutrient translocation and accumulation in seeds [16]. Additionally, carotenoid biosynthesis can be boosted by engineering key enzymes like *psy* (phytoene synthase) and *crtI* (phytoene desaturase), which have been successfully introduced in crops such as Golden Rice to enhance provitamin A content in the endosperm [14]. Tissue-specific expression using endosperm or phloem promoters helps to avoid off-target metabolic effects and improve biosynthetic efficiency.

Golden Rice, a genetically engineered rice variety enriched with vitamin A, is one of the most well-known examples of biofortification [21]. Similarly, iron-fortified beans and zinc-enriched wheat varieties have been developed to combat nutrient deficiencies in vulnerable populations. These biofortified crops offer a sustainable solution for improving global nutrition, particularly in regions where dietary diversity is limited. Recent research has further highlighted the role of biofortification in combating global malnutrition. Johnson et al. [22] reviewed the impact of biofortified crops such as vitamin A-enriched maize and iron-fortified beans, emphasizing their potential in reducing micronutrient deficiencies. Additionally, CRISPR-based interventions have targeted toxic cyanide precursors in cassava, making the crop safer and more nutritious for consumption [23].

However, biofortification must be carefully regulated to balance nutritional gains with possible unintended biochemical changes. Some concerns include potential alterations in plant metabolism, interactions with other dietary components, and consumer acceptance. Rigorous testing and transparent communication with the public are essential to ensure the safe and effective implementation of biofortified crops.

### 3.3 Impact on food authenticity

The quest for organoleptic properties often leads to the production of food products that diverge significantly from those consumed by our ancestors. This raises concerns about the authenticity of modern food varieties, particularly in the context of traditional diets and food systems that were once rich in biodiversity and nutritional quality. Gepts [24] discusses the concept of food authenticity, emphasizing that the genetic diversity of crop species has been diminished due to selective breeding practices aimed at maximizing yield and market appeal.

As a result, many contemporary food products may lack the nutritional complexity and diversity that characterized traditional diets. For instance, the widespread cultivation of a limited number of crop varieties has led to a decrease in the availability of heritage and heirloom varieties that often possess unique flavors, textures, and nutritional profiles. This loss of diversity not only affects consumer choice but also undermines the resilience of food systems, as diverse diets are important for maintaining health and well-being [24].

Furthermore, the perception of authenticity in food can influence consumer trust and preferences. Many consumers today seek foods that are perceived as “natural” or “traditional,” often associating these qualities with higher nutritional value. The disconnect between modern agricultural practices and historical food systems can thus create a challenge for producers striving to meet consumer expectations while ensuring nutritional adequacy and health benefits. Educating consumers on the benefits of biofortification and sustainable breeding techniques is essential for bridging the gap between perception and scientific advancements in food genetics.

## 4 Health implications of genetic manipulation

### 4.1 Potential risks

The introduction of genetic manipulation techniques in agriculture, while offering numerous benefits in terms of crop yield and resilience, raises significant concerns regarding potential health risks associated with genetically altered foods. The safety of genetically modified organisms (GMOs) has been a contentious topic, with various studies evaluating the long-term effects of consuming these products. Zhang et al. [6] provide a comprehensive review of the promises and problems associated with genetically modified foods, emphasizing the necessity for rigorous safety assessments to understand the potential risks better.

Safety assessments for GMOs typically involve evaluating allergenicity, toxicity, and nutritional equivalence compared to non-modified counterparts. Domingo and Giné Bordonaba [25] highlight the importance of these assessments, suggesting that despite the regulation frameworks in place, concerns remain regarding the long-term effects of GMOs on human health. Their review indicates that while most studies report no significant adverse effects, the need for continuous monitoring and long-term studies is critical to identify any potential unforeseen health implications. The lack of consensus on safety raises questions about the adequacy of current testing protocols, particularly in light of emerging genetic modification techniques like CRISPR. Additionally, the potential impact of biofortified and transgenic crops on gut microbiota and immune system responses warrants further investigation.

### 4.2 Food allergens and mechanistic links to autoimmune and hormonal disorders

Emerging evidence suggests potential links between the consumption of genetically modified foods and the rising incidence of autoimmune and hormonal disorders. While establishing a direct causal relationship is challenging, several studies indicate that the alterations in the biochemical composition of genetically modified crops could contribute to health issues.

Research has suggested that some genetically modified crops may provoke immune responses that could lead to autoimmune conditions. For instance, certain genetically engineered proteins might be perceived as foreign by the human immune system, leading to an inappropriate immune response [6]. This concern is particularly pertinent for individuals with pre-existing sensitivities or allergies, as the introduction of new proteins through genetic modification could exacerbate their conditions. Additionally, unintended epitope formation in transgenic crops could increase allergenicity risks, necessitating stricter regulatory evaluations.

Moreover, hormonal disorders have also been linked to dietary patterns influenced by the prevalence of genetically modified foods. A study by the European Food Safety Authority noted that some GM crops might produce novel compounds that could disrupt endocrine function, leading to hormonal imbalances [25]. Hormonal disorders, including reproductive health issues and thyroid dysfunction, have been on the rise, prompting calls for further investigation into the potential impacts of genetically modified foods on hormonal regulation. Specific concerns include the potential for genetically modified soybeans to influence estrogen levels and reproductive health, necessitating long-term epidemiological studies.

### 4.3 Meta-analysis of GMO safety studies

Studies assessing GMO safety largely indicate no significant adverse effects; however, long-term research remains limited. A comprehensive meta-analysis of over 100 studies suggests that while no direct evidence links GMOs to widespread health risks, variations in study methodologies and potential conflicts of interest must be accounted for in future evaluations [25]. The results highlight the necessity for ongoing surveillance, particularly with the increasing adoption of CRISPR-based modifications and biofortified crops.

The relationship between genetic manipulation in agriculture and health outcomes underscores the need for a precautionary approach. As genetic techniques continue to evolve, it is important to prioritize comprehensive safety evaluations and long-term health studies to assess the potential risks associated with the consumption of genetically altered foods. Future research should integrate clinical trials, epidemiological studies, and toxicological assessments to provide a holistic understanding of GMO safety and its implications for human health.

## 5 Socioeconomic and regulatory considerations

### 5.1 Impact on farmers and markets

While GMOs offer higher yields and improved crop resilience, they can increase dependency on biotech corporations, raising ethical concerns regarding seed monopolies. Many genetically modified seeds are patented, requiring farmers to purchase new seeds each planting season rather than saving seeds from previous harvests. This dependency can impose financial burdens, particularly on smallholder farmers in developing countries, where access to credit and agricultural inputs may be limited [7]. Additionally, GM crops often require specific pesticides or herbicides, further increasing production costs for farmers [4].

Farmers also face regulatory and trade barriers when adopting GM crops, affecting global market dynamics. Countries with stringent GMO regulations may restrict imports of genetically modified commodities, limiting export opportunities for farmers in regions with widespread GMO adoption. For example, European Union regulations on genetically modified food have led to trade disputes with major agricultural exporters like the United States and Brazil [24]. Such disparities in regulation frameworks create market segmentation and uncertainty for producers and agribusinesses operating in global supply chains [7].

Moreover, the high costs associated with developing and gaining regulatory approval for GM crops result in market dominance by a few multinational corporations, reducing competition and limiting access to agricultural innovations for smaller companies and public research institutions. Addressing these challenges requires policy interventions that promote equitable access to biotechnological advancements while ensuring fair competition in agricultural markets [5].

### 5.2 Consumer perception and labeling

Despite scientific consensus on GMO safety, public skepticism persists, driven by misinformation, ethical concerns, and the perception that genetically modified foods are unnatural. Studies indicate that many consumers prefer non-GMO or organic products due to concerns over potential long-term health risks, environmental impacts, and corporate control over the food supply [6,24]. Public distrust is further exacerbated by inconsistencies in regulatory approaches and a lack of transparency in genetic modification processes [25].

Transparent labeling and public education are essential to bridge the trust gap between consumers and the biotech industry. Mandatory labeling policies for genetically modified foods vary globally. The European Union requires strict labeling of GMO-containing products, while the United States implemented the National Bioengineered Food Disclosure Standard in 2022, which requires companies to disclose genetically modified ingredients but allows flexibility in how they do so (e.g., QR codes, text, or symbols) [25]. Such variations contribute to consumer confusion and reinforce the need for standardized, clear, and accessible labeling systems [21].

Beyond labeling, proactive engagement with consumers through science communication, public outreach programs, and participatory decision-making processes can help build confidence in genetic technologies. Ensuring that consumers have access to unbiased, science-based information about genetic modification will allow them to make informed choices about their food consumption [8,3].

### 5.3 Global regulations and trade policies

GMO policies vary widely, from strict EU regulations to widespread adoption in the United States and Brazil. While some countries embrace genetic modification as a means to enhance food security and agricultural productivity, others impose strict controls due to environmental and ethical concerns. This regulatory divergence impacts international trade, creating barriers for exporters of genetically modified crops [1].

International harmonization of safety protocols and ethical guidelines is important for equitable trade and food security. Recent regulatory shifts reflect a growing acceptance of genetic technologies in agriculture. In China, approvals for multiple GM and genome-edited crop varieties have been granted to enhance yields and bolster food security [26]. Similarly, the approval of drought-resistant HB4 wheat in the United States marks a significant step toward integrating climate-resilient genetically modified crops into global markets [27]. These regulatory advancements underscore the need for harmonized safety protocols to facilitate international trade while addressing ethical and environmental concerns.

The Cartagena Protocol on Biosafety, an international agreement under the Convention on Biological Diversity, seeks to regulate the transboundary movement of GMO, ensuring that countries can make informed decisions about GMO imports based on risk assessments [24]. However, implementation remains uneven, with some nations imposing outright bans on GMOs while others adopt more permissive policies [7].

Trade agreements increasingly incorporate GMO-related provisions, shaping market access for genetically modified commodities. For instance, disputes between the United States and the European Union over GMO trade policies have highlighted the complexities of aligning regulatory standards across different jurisdictions [25]. Establishing globally accepted safety assessment frameworks and regulatory cooperation mechanisms would facilitate trade while addressing public concerns about genetic engineering in food production [4].

Furthermore, regulation policies must balance innovation with precautionary measures. Emerging genetic technologies, such as CRISPR-based genome editing, challenge existing regulation frameworks, as they may not involve the insertion of foreign DNA in the same way as traditional GMOs. Some countries, including Japan and the United States, have adopted more lenient regulatory approaches for gene-edited crops, while others, such as the European Union, classify them under the same stringent regulations as GMOs [3]. Standardizing risk assessment protocols for novel genetic techniques will be essential for fostering international collaboration and ensuring that regulatory policies keep pace with scientific advancements [8].

Ultimately, balancing economic interests, consumer rights, and environmental sustainability in GMO regulation requires global dialogue and cooperation. Policy frameworks must be adaptive, transparent, and inclusive, addressing both scientific and societal concerns to create a fair and sustainable agricultural biotechnology landscape.

The European Union has implemented a rigorous and comprehensive legal framework to regulate the authorization, labeling, and traceability of GMOs and products derived from them. Specifically, Regulation (EU) 1829/2003 establishes a unified procedure for the authorization of GM food and feed, requiring a thorough risk assessment to be conducted by the European Food Safety Authority (EFSA) prior to approval. Furthermore, Regulation (EU) 1830/2003 sets out a system for the traceability and labeling of GM products at all stages of production and distribution, thereby strengthening consumer choice and market transparency. The EFSA plays a key role in this process by providing independent, science-based risk assessments that consider health, environmental, allergenic, and nutritional aspects of each application. Together, these measures reflect the European Union’s rigorous approach to biosafety, assuring consumers of a high level of health and environmental protection while maintaining fairness and openness in the food chain.

## 6 Sustainable practices in food production

The urgent need for sustainable practices in food production has become increasingly recognized, particularly in light of global challenges such as climate change, population growth, and the depletion of natural resources. The application of sustainability in food genetics is important in ensuring that agricultural practices can meet the demands of a growing population without compromising the environment or public health. Kershen [7] underscores the legal and developmental perspectives surrounding genetically modified crops, emphasizing that sustainable agriculture must balance productivity with ecological integrity.

From a molecular biology standpoint, improving sustainability in crop production relies on precise manipulation of stress-responsive genes and pathways. Transcription factors such as *DREB1A* (dehydration responsive element binding) and members of the *NAC* family (*OsNAC10*, *SNAC1*) activate stress-inducible genes that regulate osmolyte accumulation, ABA signaling, and reactive oxygen species scavenging. Overexpression of *DREB1A* in rice and Arabidopsis enhances tolerance to drought and cold by inducing LEA proteins and detoxification enzymes [17], while *OsNAC10* has been shown to improve root architecture and drought resilience in rice, leading to increased yield under water-limited conditions [18]. Additional targets include aquaporins (*PIP2;1*), heat shock proteins (*HSP70*), and other protective molecules that stabilize cell membranes and proteins during stress [19]. These molecular mechanisms provide foundational tools for the development of climate-resilient crops through transgenic and genome editing approaches.

Kershen [7] argues that sustainable agricultural practices should prioritize the development of crops that can thrive in diverse environments while minimizing the use of chemical inputs. This involves creating a regulation framework that supports innovation and encourages the adoption of genetically engineered crops that are designed for sustainability, such as those with increased drought tolerance or reduced pesticide requirements. By fostering an environment where sustainable genetic practices are the norm, we can enhance food security and protect the ecological balance. Additionally, precision agriculture and digital farming tools, including AI-driven crop monitoring and data-driven irrigation systems, can contribute to optimizing resource use while minimizing environmental impact.

Moreover, the concept of sustainability in food production goes beyond mere environmental considerations; it encompasses economic viability and social equity as well. Sustainable food production systems aim to empower local farmers, improve livelihoods, and ensure access to nutritious food for all communities. The integration of sustainable genetic practices can thus contribute to broader socio-economic goals, promoting resilience in food systems and communities [7]. Investments in agroecology, regenerative agriculture, and organic-compatible biotechnologies can enhance soil health and biodiversity while ensuring long-term agricultural productivity.

To achieve improvements in breeding practices while maintaining nutritional integrity, collaborative research efforts are essential. The integration of diverse fields, such as plant genetics, nutrition science, and environmental studies, can lead to innovative solutions that enhance crop quality and sustainability. Collaborative initiatives between academic institutions, government agencies, and the private sector can facilitate knowledge sharing and technology transfer, ultimately leading to the development of more resilient and nutritionally rich crop varieties.

An emerging frontier in genetic research is the integration of artificial intelligence (AI) with CRISPR-based editing. AI-driven models have accelerated target gene identification and mutation predictions, streamlining the breeding process [28]. These innovations enable more precise crop modifications, reducing trial-and-error breeding cycles while optimizing traits such as drought tolerance and pest resistance.

One approach is to establish interdisciplinary research programs that focus on identifying genetic traits linked to both organoleptic qualities and nutritional value. By leveraging advanced techniques like genome editing and MAS, researchers can develop crops that not only meet consumer preferences but also contribute to improved health outcomes. For example, breeding programs could target the enhancement of essential nutrients in staple crops, thereby addressing issues of malnutrition while satisfying market demands for flavor and appearance. Genomic selection, coupled with machine learning algorithms, can further refine breeding strategies by predicting the best genetic combinations for yield stability and climate adaptability.

Furthermore, involving stakeholders such as farmers, consumers, and nutritionists in the research process can ensure that the developed varieties are relevant and beneficial to the communities they serve. Engaging these stakeholders can provide valuable insights into local food preferences, dietary needs, and agricultural practices, leading to more targeted and effective breeding strategies. Public–private partnerships can play an important role in funding sustainable agricultural research and accelerating the commercialization of climate-smart crops that align with both environmental and consumer needs.

Sustainability in food production also requires addressing food waste and improving supply chain efficiency. Biotechnological solutions, such as gene-edited crops with extended shelf life and improved resistance to post-harvest losses, can significantly reduce food wastage. Additionally, innovations in biodegradable packaging and alternative protein sources (e.g., plant-based and lab-grown meat) contribute to the sustainability of the global food system.

Prioritizing sustainable practices in food genetics and fostering collaborative research efforts are important for the future of food production. By aligning agricultural innovation with sustainability and nutritional integrity, we can create a food system that meets the needs of the present without compromising the ability of future generations to thrive. Ultimately, integrating biotechnological advancements with environmentally responsible farming practices is key to ensuring food security while preserving natural ecosystems for long-term agricultural resilience.

## 7 Integrating research

To improve breeding practices while maintaining nutritional integrity, it is imperative to promote collaborative research efforts across multiple disciplines. Interdisciplinary approaches can lead to the development of crop varieties that are not only high yielding but also nutritionally rich and sustainable. Such collaboration can involve partnerships among universities, agricultural research institutes, private companies, and government bodies, fostering an environment where knowledge and resources are shared effectively.

One key area for collaborative research is the integration of genetic improvement techniques with nutritional science. For example, transgenic approaches have been used to introduce biofortified traits into crops, such as Golden Rice enriched with vitamin A and iron-fortified wheat. These innovations help combat micronutrient deficiencies in vulnerable populations. Researchers can utilize genomic tools, such as CRISPR and MAS, to enhance the nutritional profiles of staple crops without compromising their agronomic performance. By focusing on traits that improve vitamin, mineral, and antioxidant content, breeding programs can directly address nutritional deficiencies in populations reliant on these crops [3,8].

Additionally, engaging local communities and stakeholders in the research process is important for ensuring that the developed crop varieties are relevant and beneficial. This participatory approach can help identify local food preferences, cultural dietary needs, and agricultural challenges, leading to breeding strategies that align with the realities of specific regions. For instance, involving farmers in trials can provide practical insights into the adaptability and performance of new varieties under local conditions, thereby increasing their acceptance and adoption (Table 2).

**Table 2:** Key research areas in food genetics and improvement

Research area	Key objectives	Techniques applied
Transgenic crops	Improve yield, resistance, and nutrition	Genetic engineering (Bt crops, Golden Rice)
Biofortification	Enhance micronutrient content	Gene editing, conventional breeding
Allergen reduction	Minimize food allergenicity	RNAi, CRISPR
Sustainable farming	Reduce environmental impact	MAS, precision agriculture

Ultimately, the integration of research efforts that prioritize sustainability and nutritional integrity is essential for advancing food production practices. By fostering collaboration among scientists, policymakers, and communities, we can develop innovative solutions that ensure food security while preserving ecological health and promoting public well-being.

## 8 Conclusion

### 8.1 Summarizing key points

The exploration of food genetics reveals a complex landscape characterized by both potential benefits and significant dilemmas. Advances in genetic techniques, including MAS, genome editing, transgenic modifications, and biofortification, have the power to enhance crop quality, yield, and resilience. However, as highlighted throughout this discussion, these benefits come with challenges regarding nutritional quality, food authenticity, food allergens, and potential health risks. The emphasis on organoleptic properties often overshadows nutritional considerations, raising concerns about the overall integrity of the food supply. Moreover, the implications of genetic manipulation on human health – particularly concerning autoimmune and hormonal disorders – underscore the necessity for thorough safety assessments and long-term studies.

Sustainability emerges as a pivotal theme, necessitating a shift toward practices that not only meet the demands of a growing population but also preserve the environment and public health. The integration of sustainable methods in food genetics, supported by robust legal frameworks, can foster innovation while ensuring that agricultural advancements align with ecological and societal goals.

### 8.2 Call to action

As we navigate the intricacies of food genetics, it is essential to foster ongoing dialogue among scientists, policymakers, and consumers. Collaborative efforts should focus on prioritizing health, sustainability, and transparency in food production systems. This dialogue is important in developing regulation frameworks that balance innovation with safety and sustainability, ensuring that advancements in food genetics benefit all stakeholders.

Consumers must also be engaged in this conversation, as their preferences and concerns play a significant role in shaping agricultural practices. Education and awareness initiatives can empower consumers to make informed choices about the foods they consume and advocate for transparency in food labeling and production practices.

The future of food production hinges on our ability to address the dilemmas posed by genetic techniques while prioritizing health and sustainability. By working together, we can build a resilient and equitable food system that not only meets our current needs but also preserves the planet for future generations.
